# A preliminary assessment of vital-signs-integrated patient-assisted intravenous opioid analgesia (VPIA) for postsurgical pain

**DOI:** 10.1186/s12871-020-01060-4

**Published:** 2020-06-08

**Authors:** Ban Leong Sng, Daryl Jian’an Tan, Chin Wen Tan, Nian-Lin Reena Han, Rehena Sultana, Alex Tiong Heng Sia

**Affiliations:** 1 Department of Women’s Anaesthesia, KK Women’s and Children’s Hospital, 100 Bukit Timah Road, Singapore, Singapore; 2 Anaesthesiology and Perioperative Sciences Academic Clinical Program, Duke-NUS Medical School, 8 College Road, Singapore, Singapore; 3 Division of Clinical Support Services, KK Women’s and Children’s Hospital, Singapore, Singapore; 4 Centre for Quantitative Medicine, Duke-NUS Medical School, 8 College Road, Singapore, Singapore

**Keywords:** Infusion pump, Postoperative pain, Vital sign monitoring, Oxygen desaturation

## Abstract

**Background:**

We developed a Vital-signs-integrated Patient-assisted Intravenous opioid Analgesia (VPIA) analgesic infusion pump, a closed-loop vital signs monitoring and drug delivery system which embodied in a novel algorithm that took into account patients’ vital signs (oxygen saturation, heart rate). The system aimed to allow responsive titration of personalized pain relief to optimize pain relief and reduce the risk of respiratory depression. Moreover, the system would be important to enable continuous monitoring of patients during delivery of opioid analgesia.

**Methods:**

Nineteen patients who underwent elective gynecological surgery with postoperative patient controlled analgesia (PCA) with morphine were recruited. The subjects were followed up from their admission to the recovery room/ ward for at least 24 h until assessment of patient satisfaction on the VPIA analgesic infusion pump.

**Results:**

The primary outcome measure of incidence of oxygen desaturation showed all patients had at least one episode of oxygen desaturation (< 95%) during the study period. Only 6 (31.6%) patients had oxygen desaturation that persisted for more than 5 min. The median percentage time spent during treatment that oxygen saturation fell below 95% was 1.9%. Fourteen (73.7%) out of 19 patients encountered safety pause, due to transient oxygen desaturation or bradycardia. The patients’ median [IQR] pain scores at rest and at movement after post-op 24 h were 0.0 [2.0] and 3.0 [2.0], respectively. The average morphine consumption in the first 24 h was 12.5 ± 7.1 mg. All patients were satisfied with their experience with the VPIA analgesic infusion pump.

**Conclusions:**

The use of VPIA analgesic infusion pump, when integrated with continuous vital signs monitor and variable lockout algorithm, was able to provide pain relief with good patient satisfaction.

**Trial registration:**

This study was registered on clinicaltrials.gov registry (NCT02804022) on 28 Feb 2016.

## Background

More than 230 million major surgeries are performed annually in the world that could result in moderate to severe post-surgical pain [1]. Patient-controlled analgesia (PCA) with an opioid pump is often the commonly used technique to relief pain. Opioids adverse effects such as nausea, vomiting, sedation and respiratory depression may occur especially in high risk patients. The risk of opioid-induced respiratory depression was significantly increased in the patients with advanced age, respiratory disease and obstructive sleep apnoea [2, 3], leading to the increased length of stay and overall costs [4]. Intermittent monitoring measures were highly labor intensive, yet not reliably recognized opioid-induced respiratory depression in the postoperative period [5]. According to Anaesthesia Patient Safety Foundation, patients having vital signs charted every 4 h were usually left unmonitored > 90% of the time. Since these patients were commonly administered with supplemental oxygen, this might eventually complicate the monitoring by masking hypoventilation, causing the signs of respiratory depression to be recognized only in its later stage [6].

Our overall aim was to develop a Vital-signs-integrated Patient-assisted Intravenous opioid Analgesia (VPIA) analgesic infusion pump with closed-loop vital signs monitoring and drug delivery system which embodied a novel algorithm that accounted for patients’ vital signs (oxygen saturation, heart rate). In this preliminary study, our primary aim was to investigate the incidence of oxygen desaturation (defined as oxygen saturation < 95% in a patient for more than 60 s) in post-operative patients using our VPIA analgesic infusion pump. The side effects of opioid administration (nausea, vomiting and sedation), patients’ satisfaction and vital signs monitoring data were also evaluated.

## Methods

This study was approved by the SingHealth Centralized Institutional Review Board, Singapore (SingHealth CIRB Ref: 2015/3062), and registered on Clinicaltrials.gov (NCT02804022). Written informed consent was obtained from every patient before any study procedure. The study period was between January 2017 and June 2017 and was conducted at KK Women’s and Children’s Hospital, Singapore.

We recruited female patients aged 21 to 70 years old with American Society of Anesthesiologists (ASA) status I or II, undergoing elective surgery and intending to use postoperative PCA with morphine for postoperative analgesia. The exclusion criteria were the patients with allergies to morphine, history of significant respiratory disease or obstructive sleep apnea, unwilling to wear oxygen saturation monitoring devices throughout the study duration, pregnancy and unable to comprehend the use of PCA. The recruitment was performed either in the pre-operative assessment clinic or on the same day of surgery if they had not attended pre-operative assessment clinic. An information brochure describing the use of VPIA analgesic infusion pump for post-operative analgesia, including potential side effects and complications was provided to the patients.

### Setting up of infusion pump

**Fig. 1:**
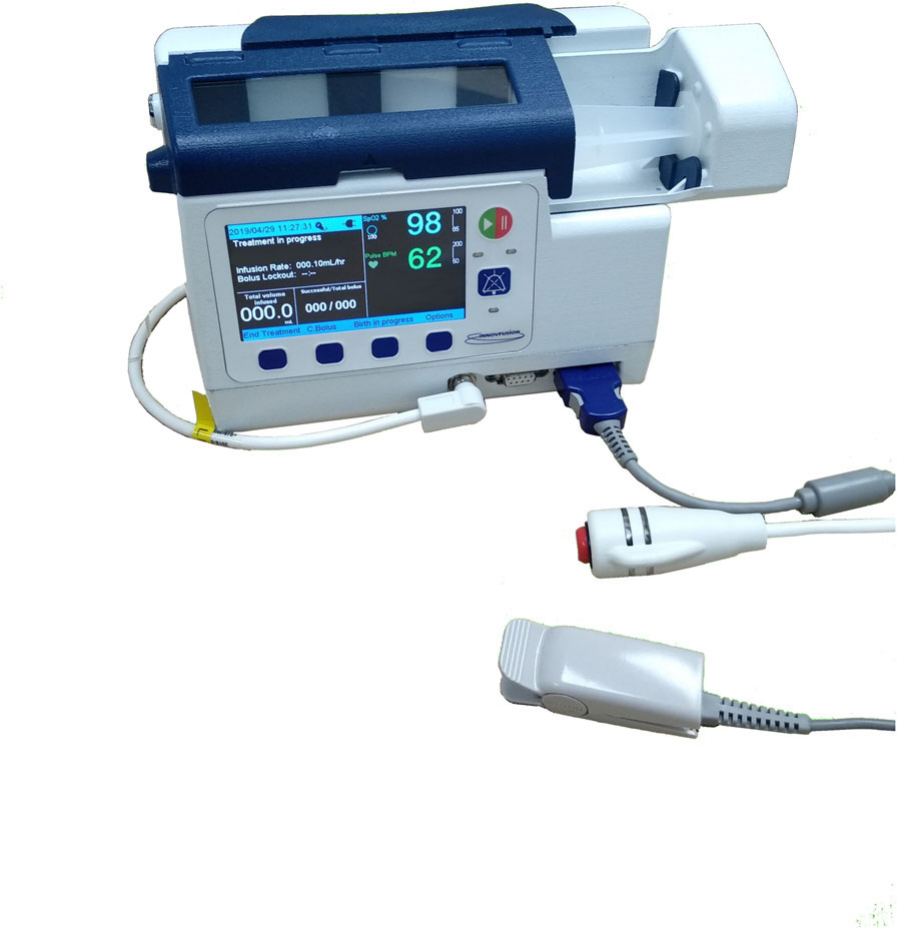
An illustration of Vital-signs-integrated Patient-assisted Intravenous opioid Analgesia (VPIA) analgesic infusion pump. The algorithm and the VPIA analgesic infusion pump (“Intellifuse pump”; Model: Opiva) was designed by Innovfusion Pte Ltd., Singapore. The written permission has been given for publication by Innovfusion Pte Ltd., Singapore

The algorithm and the VPIA analgesic infusion pump (“Intellifuse pump”; Model: Opiva) was designed by Innovfusion Pte Ltd., Singapore (Fig. 1). Intravenous morphine used in the VPIA analgesic infusion pump was administered according to the institutional guidelines: morphine diluted in normal saline to a concentration of 1 mg/ml, with bolus doses of 1 mg morphine delivered as per patient demand. In the VPIA analgesic infusion pump, vital signs monitoring (oxygen saturation, pulse rate) was programmed into the VPIA variable lockout algorithm, in which a temporary pause to the pump was triggered when vital signs safety threshold was breached; and subsequently the lockout interval was increased upon re-starting thereby improving the safety of intravenous morphine administration. That means when the vital signs were within normal range, the system was able to increase or decrease the lockout interval according to the analgesic needs of the patient. This lockout interval was a safety mechanism that limited the frequency of demands. By allowing an adequately long interval between each dose, the patients were given sufficient time to achieve the opioid’s effects before the next dose. However, if the interval were prolonged, the effectiveness of patient controlled analgesia would be reduced.

**Fig. 2:**
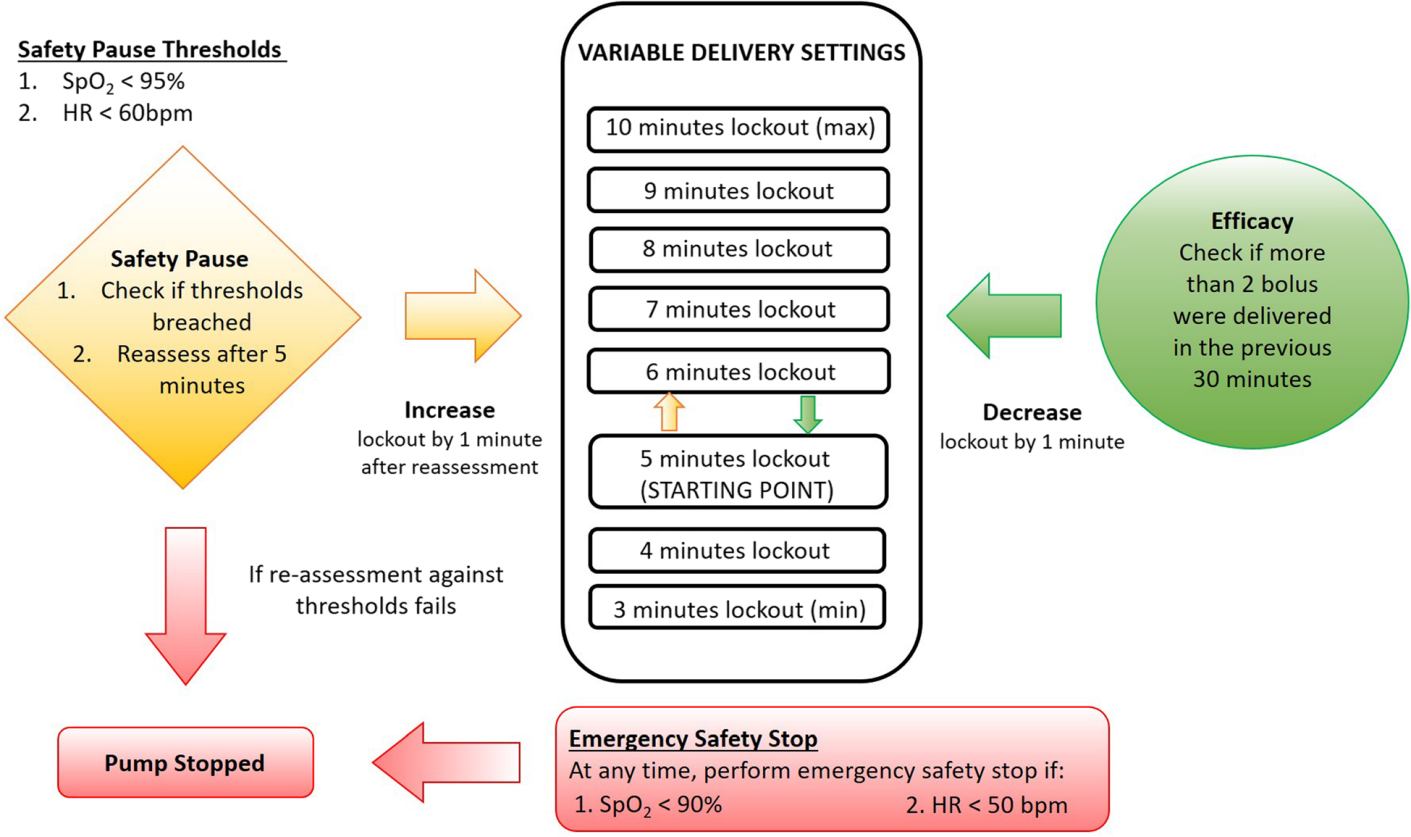
The proposed Vital-signs-integrated Patient-assisted Intravenous opioid Analgesia (VPIA) analgesic infusion pump and the variable lockout algorithm

The detailed VPIA variable lockout algorithm was illustrated in Fig. 2. The bolus lockout interval was empirically set at 7 min, and was adjusted automatically according to the patient successful demands and the patient safety in the event of abnormal vital signs monitoring. The monitoring data was performed by taking average epochs of 15 s to summarize the vital signs. Missing vital signs were dropped from the analysis. However, if there was no available vital sign for the whole duration of each epoch, a safety pause would be triggered. The VPIA variable lockout algorithm reassessed for the recovery of the patient’s’ vital signs at the end of the safety lockout period. If the patient’s vital signs did not recover to the safe levels, the pump automatically raised the on-board alarm. Conversely, if the patient’s vital signs recovered beyond the threshold limits by the end of the safety lockout period, the lockout interval would be prolonged. If at any time, there were critically abnormal vital sign parameters, the system would trigger the “emergency safety stop” function to cease the patient’s boluses. The system would be manually restarted by a clinician or a nurse after reviewing the patient.

All patients had established intravenous access before surgery. The patients were instructed on the use of the VPIA analgesic infusion pump prior to the study and educated to press the demand button whenever they needed pain relief. While in the recovery room after surgery, the VPIA analgesic infusion pump was secured with a 50 ml syringe filled with1 mg/ml morphine that was connected to the patient’s intravenous line for analgesia. The patient’s oxygen saturation and heart rate was continuously monitored by the VPIA analgesic system at least for 24 h after surgery.

### Data collection

We collected and analyzed three sets of data: (1) patient demographic, surgical and anesthetic characteristics; (2) VPIA analgesic infusion pump data including opioid consumption, patient demands, successful demands, pattern of demands, oxygen saturation and heart rate; and (3) VPIA analgesic infusion pump user feedback survey that was conducted at the end of the study.

Following the initiation of VPIA analgesic infusion pump, attending nurses who were educated on the usage and side effects of morphine therapy would monitor the patient at regular intervals and document pain scores (0–10 numeric rating scale), blood pressure, heart rate, oxygen saturation and sedation score (0 for “awake, alert”, 1 for “occasionally drowsy, easy to rouse, responds to calling”, 2 for “occasionally drowsy, difficult to rouse, responds to shaking only”; 3 for “unresponsive and unarousable-- defined as no response to voice or physical stimulation”; D for “distressed -- defined as awake and in great pain”). Side effects such as nausea and vomiting were also recorded.

An independent observer would assess the patient during the period she was placed on the VPIA analgesic infusion pump. The patient’s overall satisfaction (numerical score between 0 and 100%) with the postoperative analgesia provided, the feedback on pain relief effectiveness and any side effect from the therapy were also gathered. Once the indications for PCA opioid for pain management were deemed unnecessary by the patient’s primary care team, the VPIA analgesic infusion pump was disconnected.

### Sample size calculation and statistical analysis

The primary outcome measure of the study was the incidence of oxygen desaturation (defined as oxygen saturation < 95% in a patient for more than 60 s) in the patients using the VPIA analgesic infusion pump. The secondary outcome measures were bradycardia, sedation, nausea/vomiting, pain scores, total consumption of morphine, patient’s satisfaction score. Patient’s oxygen saturation and heart rate were measured every minute for at least 24 h. Assuming that a patient had < 3% oxygen desaturation, 0.005 width of interval and 95% confidence interval, we would require 18,000 oxygen saturation readings. Each patient would provide at least 1200 readings. Therefore, the study was adequately powered for 18 patients with 1200 readings (= 18 X 1200 ~ 21,600) for both primary and secondary aims.

The patient demographics, surgical and anesthetic characteristics were summarized as frequency with corresponding proportion, as mean ± standard deviation (SD) or median [range], whichever applicable. The incidence rate and 95% confidence intervals (CI) of binary outcomes (such as desaturation, bradycardia) were estimated based on the exact method by Wilson [7], which demonstrated to have good statistical properties even for small number of subjects and/or extreme probabilities [8, 9]. Significance level was set at 0.05 and all tests were two-tailed. SAS version 9.3 software (SAS Institute; Cary, North Carolina, USA) was used for the analysis.

## Results

Nineteen patients were recruited for this study over a 6-month period (January 2017 to June 2017), with their baseline and demographic characteristics shown in Table 1. The mean age of patients was 51.5 ± 8.8 years (range 36–66 years), the average body mass index (BMI) was 24.7 ± 4.4 kg/m^2^. All patients recruited went through scheduled open surgery, with the majority (n = 15) under total abdominal hysterectomy and bilateral salpingo-oophorectomy (TAHBSO). The rest were open myomectomy (n = 2) and salpingo-oophorectomy (n = 2). Intraoperative morphine and fentanyl was administered with a mean dosage of 8.7 ± 1.3 mg and 102.0 ± 35.3 μg respectively.

**Table 1:** Baseline and demographic characteristics of recruited subjects

Parameters	No. of patients	Mean (SD)/ Percentage
Age; years	19	51.5 (8.8)
Race	19	
Chinese	13	68.4%
Indian	3	15.8%
Malay	2	10.5%
Others	1	5.3%
Weight; kg	19	62.0 (9.9)
BMI; kg/m^2^	19	24.7 (4.4)
ASA Status	19	
I	5	26.3%
II	14	73.7%
Intraoperative morphine (mg)	19	8.7 (1.3)
Intraoperative fentanyl (mcg)	19	102.0 (35.3)

SD: Standard deviation

The patients were offered pain relief via VPIA analgesic infusion pump once they were transferred to the Post-Anesthesia Care Unit (PACU). The primary outcome measure of incidence of oxygen desaturation (Table 2) showed that all patients had at least one episode of oxygen saturation (SpO_2_) below 95% transiently during the study period. Only 13 (68.4, 95%CI 46.0–84.6%) patients had oxygen desaturations that persisted for more than 60 s. However, only 8 (42.1, 95%CI 23.1–63.7%) and 6 (31.6, 95%CI 15.4–54.0%) patients had persisted oxygen desaturation for 3 and 5 min, respectively. The median total time spent and the percentage of time that SpO_2_ fell below 95% was 35.3 min and 1.9%, respectively. During the 1st 4 h post-surgery, the median time period and the percentage of time that SpO_2_ fell below 95% was 2.08 min and 0.87%, respectively; whereas the median time spent and the percentage of time that SpO_2_ fell below 95% was 26.8 min and 1.4%, respectively after 4 h until the removal of VPIA infusion pump.

**Table 2:** The characteristics of oxygen saturation and heart rate in recruited subjects

Parameters	No. of patients	
**Incidence of oxygen desaturation**		**Percentage % (95% CI)**
At least one episode SpO2 < 95%	19	100
SpO2 < 95% persisted for > 60 s	13	68.4 (46.0–84.6)
SpO2 < 95% persisted for > 3 min	8	42.1 (23.1–63.7)
SpO2 < 95% persisted for > 5 min	6	31.6 (15.4–54.0)
		**Median [IQR]**
Post-surgery 0 h until the removal of pump		
Total time spent of SpO2 < 95%, min		35.3 [6.8–73.8]
The % of time of SpO2 < 95%		1.9 [0.4–4.2]
0–4 h post-surgery		
Total time spent of SpO2 < 95%, min		2.1 [0.2–9.2]
The % of time of SpO2 < 95%		0.87 [0.07–3.8]
> 4 h post-surgery		
Total time spent of SpO2 < 95%, min		26.8 [2.7–68.8]
The % of time of SpO2 < 95%		1.4 [0.3–3.4]
**Incidence of bradycardia**		**Percentage % (95% CI)**
At least one episode of HR < 60/min	19	100
HR < 60/min persisted for > 60 s	3	15.8 (5.5–37.6)
HR < 60/min persisted for > 3 min	1	5.3 (1.0% - 24.6)
HR < 60/min persisted for > 5 min	1	5.3 (1.0% - 24.6)

All patients had at least one episode of heart rate (HR) < 60/min with 3 (15.8, 95%CI 5.5–37.6%) patients experiencing their HR < 60/min for longer than 60 s duration (Table 2). The median time period and the percentage of time that HR fell below 60/min was 1.2 min and 0.13%, respectively. Only 1 (5.3, 95%CI 1.0–24.6%) patient had HR < 60 for both 3 and 5 min. There was no clinically significant respiratory event of note.

Fourteen out of 19 (73.7%) patients encountered the safety pauses, whereas ten (52.6%) patients had experienced an emergency safety stop during the study period. The median [range] number of safety pause was 5 [1–21]. The reasons for the safety pause included either the occurrence of oxygen desaturation or bradycardia. Eleven (57.9%) patients had the safety pause due to SpO_2_ < 95% but all had HR > 60/min during the episode. Six (31.6%) patients had HR < 60/min but all had SpO_2_ > 95% during the episode. There were 2 (10.6%) patients experiencing oxygen desaturation < 90% for more than 1 min. The median number of demands of bolus was 21 per patient. Post-operative side effects included nausea / vomiting (6/19) and pruritus (1/19).

The average morphine consumption during the stay was 3.6 ± 3.0 mg (Table 3), whereas the last pain score before sending to ward was 3 [0–6]. Minimal to moderate sedation was observed in 15 patients, whereas 4 other patients exhibited no sedation, having a median of overall sedation scoring of 1 [0–2]. Only one patient showed nausea or vomiting during this period. At 12 h post-surgery, the pain score at rest and movement in 19 patients was 2 [0–6] and 5 [0–10], respectively (Table 3). At 24 h post-surgery, patients had pain scores of 0 [0–7] and 3 [0–8] at rest and movement, respectively. The average morphine consumption in the first 24 h was 12.5 ± 7.1 mg.

**Table 3:** Pain characteristics during Post Anesthesia Care Unit (PACU) and ward stay

Parameters	No. of patients	Mean (SD) / Median [range] / Percentage
**During PACU stay**	19	
Morphine (mg)	19	3.6 (3.0)
Last pain score before discharge (0–10)	19	3 [0–6]
Last sedation score before discharge (0–3)	19	1 [0–2]
Nausea/vomiting before discharge (0–3)	19	0 [0–1]
During Ward stay		
**12 h Post-op**	19	
Pain score (at rest) (0–10)	19	2 [0–6]
Pain score (movement) (0–10)	19	5 [0–10]
Sedation score (0–3)	19	0 [0–1]
Nausea/vomiting (0–3)	18	0 [0–3]
**24 h Post-op**	19	
Pain score (at rest) (0–10)	19	0 [0–7]
Pain score (movement) (0–10)	19	3 [0–8]
Sedation score (0–3)	19	0 [0–1]
Nausea/vomiting (0–3)	19	0 [0–1]
Morphine consumption (24 h; mg)	19	12.5 (7.1)
Side effect		
Nausea / vomiting	6	31.6%
Pruritus	1	5.3%

User feedback received on VPIA analgesic infusion pump showed that all patients agreed that the pump was safe and effective to use, although they remained neutral on the mobility of the pole where the pump was mounted onto (Table 4).

**Table 4:** Post-operative feedback (n = 19) on the VPIA analgesic infusion pump

Parameters	Values [range]
Feedback (1–5; 1: Strongly disagree; 5: strongly agree)	
Patient handset button	4 [4–5]
Mobility of pole with mounted pump	3 [3–4]
No interference of vital signs monitoring with treatment	4 [1–5]
Pump safety	4 [3–5]
Pump effectiveness	4 [3–5]

## Discussion

The results of this preliminary assessment on our novel VPIA analgesic infusion pump suggested that the use of this drug delivery system, when integrated with continuous physiological monitoring and a variable lockout algorithm, was able to provide pain relief with good patient satisfaction in post-operative acute pain management. No significant adverse event was observed in this study.

Several studies evaluated the incidence of oxygen desaturation after different types of analgesia, but few analyzed the oxygen saturation continuously for 24–60 h after surgery [10]. Our results showed that all patients had at least one episode of oxygen desaturation (SpO_2_ < 95%) transiently, whereas only 13 (68.4%) patients had persistent oxygen desaturations more than 60 s. Moreover, only 8 (42.1%) and 6 (31.6%) patients had persisted oxygen desaturation for 3 and 5 min, respectively. It was important to understand the percentage of time spent with oxygen desaturation. Motamed et al. [11] demonstrated that the time spent with SpO_2_ < 95% was about 65 and 40% during 1st and 2nd postoperative night, respectively. This was much higher than our results that showed the median time period and percentage of time spent with SpO_2_ < 95% was 35.3 min and 1.9%, respectively. The reason behind this might due to our novel VPIA analgesic infusion system that integrated with continuous vital signs monitor and a variable lockout algorithm.

Hospital practice of intermittent vital signs monitoring with opioid delivery became an increasing concern. More than 75% of patients with moderate to severe sleep apnea were undiagnosed and conventional risk stratification for heightened post-op monitoring could potentially miss the patients at increased risk of respiratory depression [12]. Respiratory depression was reported in up to 5% patients with the use of PCA opioids [5, 13]. A retrospective study on Chinese patients receiving PCA intravenous morphine showed that the incidence of respiratory depression (as defined by SpO_2_ < 90% for longer than 1 min) was 1.62%. The present study showed a higher incidence due to the difference in the definition of oxygen desaturation and the continuous monitoring system because conventional intermittent routine monitoring could under-diagnose the events of oxygen desaturation [14]. Early recognition and the detection of risk of opioid-induced respiratory depression by continuous monitoring vital signs could mitigate the chance of patients’ increased length of stay, the risk of hospital-acquired infections and the increased costs [15].

This study concentrated on varying the duration of the lockout interval as a safety mechanism. This safety of margin allowed an interval to be long enough for the drugs to take effect in the patients from the present bolus dose before the next bolus was delivered. There was no recommended optimal lockout interval, hence this would need to be individualized to the patient’s pain relief and safety requirements [16]. Our results showed that the morphine consumption was 12.5 mg in 24 h after surgery which was comparable with other groups. Chou et al. found that the average morphine consumption of 40 mg in postoperative 48 h in 80 patients that underwent total abdominal hysterectomy and later [17]. Another study on patients after total knee arthroplasty showed an average morphine consumption of 27 mg in 48 h, indicating the different opioid consumptions across various surgical procedures [18].

We also gathered the patient-centered outcomes through the patients’ feedback on the device setup (patient handset, pole stand) and their overall treatment experiences (the interference with treatment, the safety and the effectiveness). The overall feedback was positive but the patients remained neutral on the reduced mobility of the pump due to the integration of the vital signs monitor. Future design of a more portable setup would be desirable.

This preliminary study was an initial assessment of our newly developed VPIA analgesic infusion pump. The limitations of this study would include the small number of subjects conducted in only gynecological surgeries. The larger trials in patients with different demography, medical comorbidities and surgical procedures would need to be investigated. This system could be more refined with higher risk patients in future. The physical design of the pump would be reduced to enable great mobility on the patient transfer. The artefact interference with the algorithms could led to false alerts in the present system. Hence., future study plans will include refining new vital signs sensors, incorporating a respiratory rate monitoring and better integration with the delivery system to ensure better data capture.

## Conclusions

In conclusion, this preliminary assessment on the VPIA analgesic infusion pump system has found that this novel pump was able to detect and automatically respond to oxygen desaturation and bradycardia by instituting safety pause, and to provide pain relief with good patient satisfaction in post-operative acute pain management. The larger studies with adequate number of subjects would be needed to evaluate the efficiency of this drug delivery system.

## Data Availability

The datasets used and/or analysed during the current study are available from the corresponding author on reasonable request.

## Abbreviations

ASA: American Society of Anesthesiologists
BMI: Body mass index
CI: Confidence intervals
IQR: Interquartile range
PACU: Post-Anesthesia Care Unit
PCA: Patient controlled analgesia
SD: Standard deviation
SpO2: Oxygen saturation
TAHBSO: Total Abdominal Hysterectomy and Bilateral Salpingo-oophorectomy
VAS: Visual analogue scale
VPIA: Vital-signs-integrated Patient-assisted Intravenous opioid Analgesia
